# The Factor Structure and Validity of the Psychopathy Checklist‐Short Version When Used With Autistic Psychiatric Inpatients

**DOI:** 10.1002/aur.70004

**Published:** 2025-02-18

**Authors:** Kate Maguire, Magali Barnoux, Josie Collins, Clare L. Melvin, Ismay Inkson, Regi T. Alexander, John Devapriam, Conor Duggan, Lee Shepstone, Ekkehart Staufenburg, Paul Thompson, David Turner, Essi Viding, Peter E. Langdon

**Affiliations:** ^1^ School of Health and Social Care University of Essex Colchester UK; ^2^ Tizard Centre University of Kent Kent UK; ^3^ Department of Law and Criminology, Royal Holloway University of London London UK; ^4^ Department of Psychology University of East Anglia Norwich UK; ^5^ Norfolk Community Health and Care NHS Trust Norwich UK; ^6^ University of Hertfordshire Hatfield UK; ^7^ Little Plumstead Hospital, Hertfordshire Partnership University NHS Foundation Trust Norwich UK; ^8^ Herefordshire and Worcestershire Health and Care NHS Trust Worcester UK; ^9^ Division of Psychiatry and Applied Psychology, School of Medicine University of Nottingham Nottingham UK; ^10^ Norwich Medical School University of East Anglia Norwich UK; ^11^ Intellectual Disabilities Research Institute (IDRIS) University of Birmingham Birmingham UK; ^12^ Division of Psychology and Language Sciences University College London London UK; ^13^ Birmingham Community Healthcare NHS Foundation Trust Birmingham UK

**Keywords:** autism, crime, intellectual disabilities, psychiatric inpatient, psychopathy, risk

## Abstract

The Psychopathy Checklist Short Version (PCL:SV) is a brief measure of psychopathy. This study aimed to assess the reliability and validity of the PCL:SV with autistic adults detained in inpatient psychiatric care. Data were collected from 282 autistic adults at two time points separated by 12‐months. Reliability and validity were investigated using omega, regression, receiver operating characteristic curves, and correlational analysis. PCL:SV Total, Factor 1, and Factor 2 had satisfactory to high reliability and construct validity. Higher PCL:SV scores were associated with poorer treatment progress, a longer length of stay, and previous criminal offending. Factor 1 was associated with a forensic history, detention under Part III of the Mental Health Act, and a personality disorder diagnosis, while Factor 2 was also associated with the absence of a forensic history, detention under Part II of the Mental Health Act, but not a personality disorder diagnosis. It was thought that Factor 2 most likely captured data associated with autism and/or intellectual disabilities (e.g., behaviors that challenge). Those with intellectual disabilities were less likely to have convictions, a history of violent offending, or a forensic history. They were also more likely to be detained under Part II of the Mental Health Act, and were more likely to have had a positive transfer 12‐months later to a ward with lesser security. The PCL‐SV correlated as expected with the HCR‐20 and the START. This study provides preliminary evidence to support the use of the PCL:SV with autistic adults, including those with intellectual disabilities, within inpatient psychiatric hospitals.

**Summary**

- Psychopathy is a construct that is characterized by shallow emotions, lack of empathy or remorse, callousness, and poor behavioral control.
- We recruited autistic psychiatric inpatients into our study, and their clinical teams completed the Psychopathy Checklist—Short Version (PCL:SV) to see how well this measure worked with autistic adults. Our results indicated that higher PCL:SV scores were associated with poorer treatment progress, a longer length of stay, and previous criminal offending and aggressive behavior 12‐months later.

Psychopathy is a construct characterized by shallow emotions, lack of empathy or remorse, callousness, and poor behavioral control (Cleckley 1941; Hare 1991). Prevalence in the general population is estimated at 0.6% (Coid, Yang, Ullrich, Roberts, and Hare 2009), with a higher prevalence among offenders (Coid, Yang, Ullrich, Roberts, Moran, et al. 2009). It has long been associated with criminal and violent behavior and is a key predictor of recidivism (Azevedo et al. 2020; Hare 1999; Woodworth and Porter 2002).

Little is known about the co‐existence of autism and psychopathy, and both conditions are characterized by difficulties with perspective‐taking. Autistic individuals do come into contact with the criminal justice system (CJS), and prevalence estimates of criminal justice contact vary widely from 0.2% to 62.8% (Collins et al. 2022). These prevalence studies are fraught with methodological problems, and on the whole, autistic individuals present with a reduced risk of engaging in crime (Collins et al. 2022).

In an attempt to disentangle the relationship between autism and psychopathy, Rogers et al. (2006) proposed that while individuals can have both autism and psychopathy, the two are likely to be distinct and separate, which was referred to as the “double hit” hypothesis; that is, those with both autism and psychopathy have two separate conditions which represent a “double‐hit.” Subsequent genetic and experimental data appear aligned with this hypothesis (e.g., Jones et al. 2010; O'Nions et al. 2014, 2015). In their systematic review, Maguire et al. (2024) investigated the relationship between autism and psychopathy. Across the included studies, a heightened prevalence of psychopathic traits among autistic individuals or those exhibiting high autistic traits was observed compared to the general population. But, there was evidence to support the view that cognitive empathy tends to be impaired among those with autism (e.g., Jones et al. 2010; Klapwijk et al. 2016), while affective empathy tends to be impaired among those scoring high on measures of psychopathy (e.g., Lockwood et al. 2013; Oliver et al. 2016), lending weight to the argument that autism and psychopathy are distinct. Those with both autism and psychopathy presented with difficulties with both cognitive and affective empathy, which has implications for risk assessment.

Maguire et al. (2024) also pointed out that there is a lack of validated measures for identifying psychopathic traits within autistic individuals. This is relevant, as both autism and psychopathy are associated with difficulties with empathy and measures of psychopathy should capture features of psychopathy, and not autism. Incorrectly labeling an autistic person as having psychopathy will cause substantial harm. Therefore, robust methods for measuring psychopathy with autistic individuals are needed to help researchers further develop an understanding of the relationship between autism and psychopathy, while enabling clinicians to accurately assess clinical risk and develop risk mitigation strategies (Alexander et al. 2016; Barnoux et al. 2020; Melvin et al. 2017). Barnoux et al. (2020) argued that autistic individuals who have psychopathy are likely to present with an increased risk of committing crimes and require longer stays within forensic psychiatric and/or criminal justice settings due to the nature or degree of this risk. It is important that these risks and treatment needs are accurately identified among autistic people.

The Psychopathy Checklist—Revised (PCL‐R) (Hare 1991) is one measure of psychopathy which is a widely used as a risk assessment tool within the CJS (Hare 2016) but can be lengthy to complete. Therefore, the shorter Psychopathy Checklist‐Screening Version (PCL:SV) was derived (Hart et al. 1995). The PCL:SV is reported to be the strongest predictor of violence in psychiatric inpatient units when compared to other risk assessment tools (Doyle et al. 2002), and there is evidence that it predicts future violence amongst adults with intellectual disabilities (Gray et al. 2007), better than the PCL‐R (Morrissey, Mooney, et al. 2007).

There is also evidence to indicate that psychopathy is associated with increased personality disorder symptomatology (Coid and Yang 2008; Coid and Ullrich 2010), including symptoms or a diagnosis of antisocial, histrionic, and borderline personality disorder, as well as paranoid personality disorder (Bergstrøm et al. 2024). There is also evidence that autism and personality disorder may share overlapping features (e.g., Dudas et al. 2017) which may make accurate diagnosis challenging (Rinaldi et al. 2021). Considering antisocial personality disorder, Murphy (2006) reported that autistic inpatients and inpatients with schizophrenia performed more poorly on theory of mind tests than those with dissocial or borderline personality disorder within a high secure hospital, suggesting that those with autism or schizophrenia may have additional difficulties with perspective‐taking. However, little is known about the relationship between personality disorder, autism, and psychopathy.

Within the current study, to investigate the reliability and validity of the PCL:SV when used with autistic inpatients detained within psychiatric hospitals, we captured data at two time points separated by 12‐months. We initially examined whether the PCL:SV was associated with length of hospital stay, criminal history, violence offenses, forensic history, and diagnosis of personality disorder. Second, we investigated predictive validity by examining if the PCL:SV predicted: (a) moves across secure wards, and (b) aggressive or problematic behavior 12‐months later. Finally, we investigated convergent validity by determining whether the PCL:SV was associated with other measures of clinical risk.

## 1 Method

### 1.1 Transparency and Openness

The study design and analysis were not preregistered. Data, analysis code, and research materials are not available. Data were analyzed using R (R Core Team 2023) and the package ggplot, version 3.2.1 (Wickham 2016).

### 1.2 Design and Setting

This study utilized a prospective cohort design with two measurement points, separated by 12 months. Fifty‐nine inpatient hospitals across 26 NHS Trusts and 7 hospitals from independent healthcare providers in England and Wales took part in this study. Sites were comprised of 22 low secure units, 13 medium secure units, two high secure units (low, medium and high secure units vary according to physical and procedural security and are predominantly for those with convictions, or behaviors likely to be criminal), 11 assessment and treatment units (for rehabilitation, acute mental health, behavioral problems), 12 locked hospital units (for rehabilitation, acute mental health, psychiatric intensive care or step‐down services) and six open hospital units (for acute admissions, psychiatric services, and specialist residential services).

### 1.3 Participants

Clinicians were asked to provide data about all their inpatients who met our eligibility criteria. Data were captured about 282 participants, who at the time of data collection were detained under the Mental Health Act, 1983, and/or subject to the Mental Capacity Act, 2005. All participants had a diagnosis of autism, including 251 males, 30 females, and one transgender person. Age ranged from 18 to 67 years, *M* = 33.29; SD = 11.70. The majority identified as Caucasian (88.6%), followed by mixed race (5%), Black African or Black Caribbean (4%), Asian (2%), and Chinese (0.4%). At baseline enrollment, most were single (98%), four were in relationships (1%), one participant was divorced (0.4%) and the majority did not have children (98%). Data about marital status was missing for one participant. Over half the sample had attended special educational needs schools (57%) and 43% were educated in mainstream schools. Data about educational status were missing for two participants. Forty‐nine percent of the sample also had a diagnosis of intellectual disability. Regarding autism diagnosis, 47% had a diagnosis of childhood autism, 12% had a diagnosis of atypical autism, 39% had a diagnosis of Asperger syndrome, and 2% were diagnosed with pervasive developmental disorder‐not otherwise specified. Fifty‐one percent were detained under forensic sections, 44% were detained under civil sections as defined within the Mental Health Act, 1983, and a further 5% of participants were detained under the Mental Capacity Act, 2005. Data on section type was missing for one participant.

### 1.4 Eligibility Criteria

Individuals were eligible for inclusion in the study if they were aged 18 years or older, had an ICD‐10 diagnosis of an autism spectrum disorder made by a Clinical Psychologist, Psychiatrist, or other appropriately qualified professional, and were detained within a hospital using the Mental Health Act, 1983, or subject to the Mental Capacity Act, 2005. There were no specified exclusion criteria.

### 1.5 Materials and Measures

A range of file‐based information was collated from clinical records and staff working with the participants, including data on hospital admissions, an individual's detention and ward security level, behavioral factors, forensic history (a history of committing crime), and data on diagnoses of personality disorders as recorded by clinicians at the site. This information was anonymized at the site by clinicians.

### 1.6 Aggressive/Problematic Behavior

Information regarding the frequency and type of aggressive or problematic behavior exhibited by participants was recorded by clinicians over the preceding 12‐week period prior to 12‐month census date. Clinicians were asked to document all instances of aggression during this period. Recorded data were sorted into eight frequency categories, as well as “overall presence/absence” of behaviors, and a category specifying if there was any evidence of clear violent intent exhibited by the participants across these behaviors, Table 1. Data were categorized independently by two researchers and any discrepancies were discussed within the research team until consensus was reached, *k* = 1.

**TABLE 1:** Definitions of aggression or problematic behavior categories.

Physical aggression	Behaviors that lead to physical harm, such as hitting others.
Verbal aggression	Behaviors where individuals were verbally aggressive towards others, such as shouting or racial abuse.
Sexual behavior	Behaviors deemed inappropriately sexual in nature, such as masturbating in public.
Violence to self	Behaviors that led to self‐injury, such as cutting or head banging.
Rule breaking	Behaviors that violated rules of the forensic mental health setting, such as absconding.
Threats of violence/aggression	Behaviors where individuals verbally threatened others, such as threatening to kill others.
Intimidating behavior	Behaviors where participants were physically threatening others through body language, such as raising fists.
Inappropriate behavior	Behaviors not considered socially acceptable behaviors, such as spitting/public defecation.
Overall presence	Overall presence of all recorded aggressive/problematic behaviors (Y/N).
Violent intent	Was there evidence of clear violent intent for behaviors? (Y/N)

### 1.7 The Psychopathy Checklist: Screening Version

The PCL:SV is a 12‐item, two‐factor tool designed for screening psychopathic traits and behaviors across forensic and non‐forensic populations in individuals aged 16 years and older (Brazil and Forth 2016). Factor 1 assesses the interpersonal and affective features of psychopathy, such as deceitfulness, grandiosity, and lack of remorse and empathy, and Factor 2 assesses the socially deviant or antisocial behavior associated with psychopathy, such as impulsiveness and poor behavioral control. Items are scored on a three‐point scale according to lifetime presence and severity of symptoms (0 = absent, 1 = possibly or partially present, and 2 = present). Individuals scoring 18 or over are considered as “psychopathic” and those scoring 13–17 are considered “maybe psychopathic” (Hart et al. 1995).

The PCL:SV was completed by senior clinicians (e.g., psychiatrist or psychologist) with responsibility for providing inpatient care. Each senior clinician had experience of interviewing the individual patient and was familiar with their history and records, including their diagnosis. While some clinicians had prior training in the PCL‐R, all clinicians, regardless of experience, attended a 1‐day group training programme for this study, which included training in the PCL‐SV assessment procedure and practice scoring. This is not dissimilar from previous studies, including the MacArther Violence Risk Assessment Study (Skeem and Mulvey 2001).

### 1.8 Historical, Clinical, and Risk Management Tool

The HCR‐20^V3^ (Douglas et al. 2013) is a 20‐item tool to assess the risk of violence in 18–65‐year‐olds, containing three subscales: historical (10 items), clinical (5 items) and risk management (5 items), accounting for past, present, and future risk factors. Items are scored on a three‐point scale (0 = absent, 1 = possibly or partially present, and 2 = definitely present) and a final summary rating of low, moderate, or high risk for violence is given. Although no cut‐off points are provided, it is generally considered that the more risk factors present, the greater the risk. The HCR‐20^V2^ has been subject to more than 200 empirical validations demonstrating its effectiveness as a risk assessment tool (Douglas et al. 2014) and versions 2 and 3 are strongly correlated (0.69–0.90) (Douglas and Belfrage 2014). The HCR‐20^V3^ is routinely completed within secure services within England; the most recently completed version was taken from electronic patient records.

### 1.9 Short‐Term Assessment of Risk and Treatability (START)

The START (Webster et al. 2004) is a 20‐item tool used to evaluate short‐term risk of aggression, in individuals aged 16 and above with psychiatric disorders. It assesses an individual's strengths and vulnerabilities, with items rated on a three‐point scale (0 indicates no vulnerability/strength evident, 1 indicates moderate vulnerability/strength, and 2 indicates high vulnerability/strength). Raters then provide an overall risk rating (low, moderate, or high) about the likelihood of seven risk outcomes occurring: violence to others, self‐harm, suicide, substance abuse, victimization, self‐neglect, and unauthorized absence. Predictive validity of aggression has been demonstrated (Braithwaite et al. 2010; O'Shea et al. 2016). The START was also routinely completed by some included sites, and where this was the case, the most recent version was taken from electronic patient records. Where this was not the case, clinicians attended a 1‐day group training programme for this study, which included training in the START assessment procedure and practice scoring.

### 1.10 Analysis

All analyses were completed using R statistical software (R Core Team 2023). Descriptive statistics and the reliability coefficients, McDonald's Omega (*ω* =) and Cronbach's alpha (*α* =) were initially calculated. A confirmatory factor analysis was conducted on the PCL:SV items to determine if the two factor solution presented in the manual was appropriate for this sample, which was the case (Hart et al. 1995). All analyses were run using PCL:SV Total Scores, Factor 1, and Factor 2 separately due to collinearity. Data were not normally distributed, and alternative and appropriate tests were chosen.

For construct validity, regression was used to examine the relationship between the PCL:SV, length of time spent in hospital, and criminal offending. History of criminal offending was capture in two ways: (1) current convictions, cautions, and reprimands, which are a count of those associated with only the current hospital admission, and (2) total convictions, cautions, and reprimands, which are a count of all lifetime convictions, cautions, and reprimands. Negative binomial regressions were used for all count data as the assumptions governing linear and Poisson regression were violated. Age and/or diagnosis of intellectual disability (ID) were included in some models as appropriate covariates. Logistic regression models had appropriate fit and were run with the following binary outcome variables: (a) diagnosis of personality disorder (yes/no), (b) forensic background (contact with the CJS) (yes/no), and (c) Mental Health Act section (civil/forensic).

Multinomial logistic regression was used to examine the relationship between the PCL:SV and any changes in ward location (no change, transferred, discharge) at 12‐month follow‐up. Logistic regression was used to examine whether any ward transfers were positive or negative as an index of treatment progress at 12‐months. Positive change was defined as moving down a security level (e.g., from high or medium to low security). Negative change was defined as moving up a security level (e.g., from low to medium or high security) or no change in security level due to too few cases in the negative change category. McFaddon's Pseudo *R* ^2^ was reported for models as an approximation to variance explained (Harrell Jr. 2016).

Receiver operating characteristic curve (ROC) analysis was used to determine the predictive validity of the PCL‐SV for aggressive or problematic behavior over the preceding 12‐weeks at the 12‐month follow‐up. Values range from 0, a perfect negative classification, 0.5, a completely chance outcome to 1, a perfect prediction (Schmidt et al. 2006). Values below 0.70 are considered poor, 0.70–0.80 moderate, 0.80–0.90 good, and those higher than 0.90 are considered excellent (de Hond et al. 2022).

Correlations were used to measure the strength and direction of the relationship between the PCL:SV, HCR20^V3^, and START. Finally, a sensitivity analysis for missing data using multiple imputation was conducted to ensure that results were robust to missing data assumptions (see Supporting Information). Additionally, Little's multivariate test (Little 1988) indicated that data were missing completely at random and unbiased (*p* = 0.99).

### 1.11 Ethical Considerations

A favorable ethical opinion was granted by NHS Wales Research Ethics Committee 7 and associated Health Research Authority approval (REC Ref: 15‐WA‐0246; IRAS: 181659). Data were anonymized at the site prior to being shared with the research team, allowing for the continued confidentiality of participants.

## 2 Results

### 2.1 Descriptive Statistics and Reliability

Descriptive statistics for all our measures are found within Table 2. Within the sample, 13.48% (*n* = 38) met criteria for psychopathy using the cut‐off score of ≥ 18; 18.79% (*n* = 53) met criteria for “maybe psychopathic” using the cut‐off score of 13–17; the remaining 58.51% (*n* = 165) were categorized as “non psychopathic.” Comparisons between these three groups across variables are found within Table 6 in Supporting Information. PCL‐SV data were missing for 26 participants. Structural reliability estimates indicated good to excellent reliability for the PCL:SV Total Score, *ω* = 0.97, 95% CI [0.93; 1.00], and *α* = 0.87, 95% CI [0.84; 0.89]. For Factor 1, the reliability estimates were also good to excellent, *ω* = 0.93, 95% CI [0.88; 0.98], *α* = 0.83, 95% CI [0.80; 0.86]. For Factor 2, the reliability estimates were satisfactory to good, *ω* = 0.88, 95% CI [0.82; 0.93], and *α* = 0.77, 95% CI [0.72; 0.81].

**TABLE 2:** Descriptive statistics for included variables.

	*n*	*M*	SD	Min–max	Frequency
PCL:SV Total	257	10.70	5.83	0–24	—
Factor 1	257	4.81	3.23	0–12	—
Factor 2	256	5.89	3.30	0–12	—
CLoS	282	928.00	1534.23	1–17,934	—
Total days spent in SPC	271	2694.07	3332.57	7–20,805	—
Total previous CCR	271	4.46	16.96	0–235	—
Total current CCR	280	1.94	3.50	0–33	—
Total violent offenses	251	2.43	4.41	0–42	—
Forensic background	281	—	—	—	No—113 Yes—168
Diagnosis of PD	282	—	—	—	No—228 Yes—54
Diagnosis of LD	282	—	—	—	No—145 Yes—137
Mental health section	267	—	—	—	Forensic—144 Civil—123
Physical aggression	176	1.49	5.12	0–52	Present: *n* = 60 (34%)
Verbal aggression	176	1.41	2.66	0–17	Present: *n* = 82 (47%)
Sexual behavior	176	0.41	1.24	0–10	Present: *n* = 32 (18%)
Violence to self	176	0.59	3.23	0–38	Present: *n* = 26 (15%)
Rule breaking	176	0.70	2.04	0–15	Present: *n* = 51 (29%)
Threats of violence/aggression	176	0.75	2.44	0–20	Present: *n* = 40 (23%)
Intimidating behavior	176	0.95	2.86	0–22	Present: *n* = 47 (27%)
Inappropriate behavior	176	0.89	3.58	0–31	Present: *n* = 31 (18%)
Total frequency	176	7.19	12.93	0–84	Present: *n* = 126 (72%)
Violent intent	178	—	—	—	Present: *n* = 100 (56%)

Abbreviations: CCR = convictions, cautions, and reprimands, CLoS = current length of stay (days), PD = personality disorder, SPC = secure psychiatric care.

**FIGURE 1:**
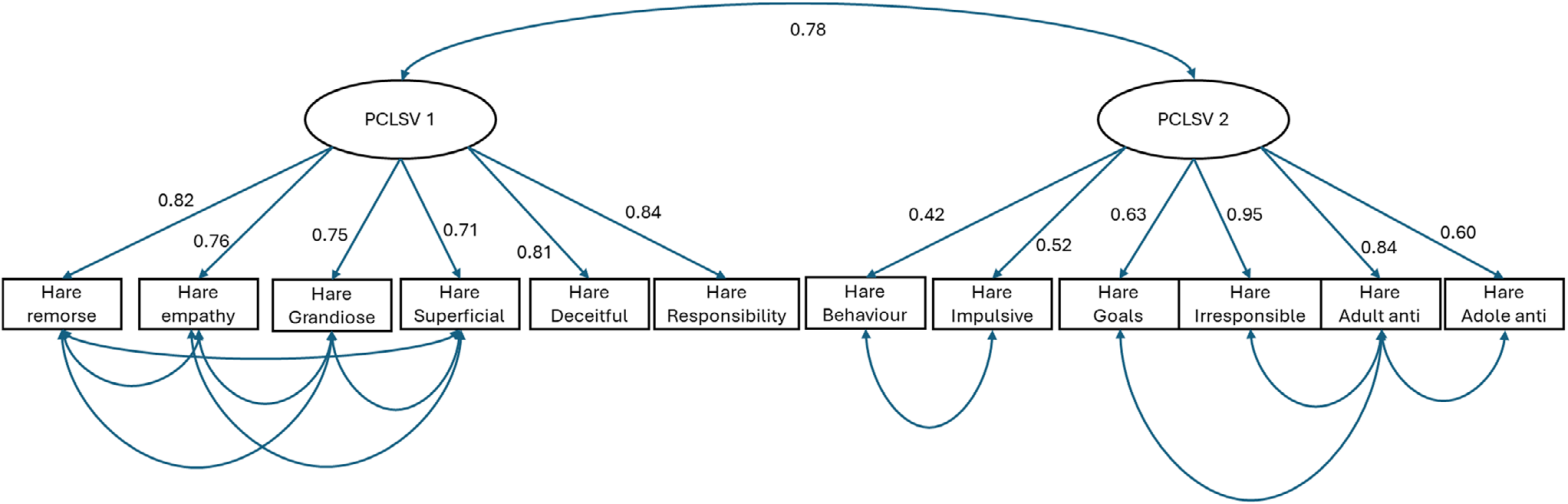
Path diagram for two factor confirmatory factor analysis.

A confirmatory factor analysis was conducted on our data to confirm that a two‐factor structure was appropriate for this sample. We found satisfactory model fit for our two‐factor model after introducing residual covariances, *χ* ^2^ (66) = 2417.16, *p* < 0.001, CFI = 0.98, TFI = 0.96, RMSEA = 0.071, and SMSR = 0.067, Figure 1.

### 2.2 Construct Validity

Controlling for age and diagnosis of intellectual disability, PCL:SV Total Score, Factor 1, and Factor 2 significantly and positively predicted current length of stay, total days spent in mental health hospitals, and total previous convictions, cautions, and reprimands, Table 3a. However, controlling for age and diagnosis of intellectual disability, PCL:SV Total Score, Factor 1, and Factor 2 did not significantly predict total *current* convictions, cautions, and reprimands; being older and having an intellectual disability was associated with fewer current convictions, cautions, and reprimands, Table 3a. PCL:SV Total Score, Factor 1, and Factor 2 significantly predicted the total number of violence offenses in the positive direction; having an intellectual disability was associated with fewer violent offenses, Table 3a. Results from missing data analysis were similar (see Supporting Information).

**TABLE 3:** (a) Negative binomial regression: The relationship between PCL:SV Total and Factor scores and length of stay and criminal offending history. (b) Logistic regression: PCL:SV and forensic background, personality disorder, and Mental Health Act section. (c) Multinomial logistic regression: PCL:SV and ward location at 12 months follow up. (d) Logistic regression: PCL:SV and changes in security ward at 12 months.

Panel (a)		*β*	SE	*z*	*p*	IRR (95% CI)	Pseudo *R* ^2^ (McFaddon)
CLoS	Model 1						0.0051
	Age	0.02	0.01	2.806	< 0.01***	1.02 [1.00–1.03]	
	PCL:SV Total	0.04	0.01	3.6	< 0.001**	1.04 [1.02–1.07]	
	Intellectual disabilities	0.12	0.13	0.87	0.38	1.12 [0.86–1.46]	
	Model 2						0.0041
	Age	0.02	0.01	2.99	< 0.01**	1.02 [1.01–1.03]	
	Factor 1	0.06	0.02	2.95	< 0.01**	1.06 [1.02–1.11]	
	Intellectual disabilities	0.17	0.14	1.22	0.22	1.18 [0.9–1.54]	
	Model 3						0.0053
	Age	0.02	0.01	2.782	< 0.01**	1.02 [1.00–1.03]	
	Factor 2	0.07	0.02	3.519	< 0.001***	1.07 [1.03–1.12]	
	Intellectual disabilities	0.09	0.13	0.65	0.52	1.09 [0.84–1.42]	
Total days spent in SPC	Model 1						0.022
	Age	0.05	0.01	10.169	< 0.001***	1.06 [1.04–1.07]	
	PCL:SV Total	0.04	0.01	3.51	< 0.001***	1.04 [1.02–1.06]	
	Intellectual disabilities	−0.09	0.13	−0.73	0.46	0.91 [0.71–1.17]	
	Model 2						0.022
	Age	0.06	0.01	10.378	< 0.001***	1.06 [1.05–1.07]	
	Factor 1	0.07	0.02	3.59	< 0.001***	1.07 [1.03–1.12]	
	Intellectual disabilities	−0.04	0.13	−0.3	0.77	0.96 [0.75–1.24]	
	Model 3						0.022
	Age	0.06	0.01	10.248	< 0.001***	1.06 [1.05–1.07]	
	Factor 2	0.06	0.02	3.07	< 0.01**	1.06 [1.02–1.10]	
	Intellectual disabilities	−0.1	0.13	−0.77	0.44	0.91 [0.71–1.16]	
Total previous CCR	Model 1						0.036
	Age	0.03	0.01	2.841	< 0.01**	1.03 [1.01–1.06]	
	PCL:SV Total	0.12	0.02	4.921	< 0.001***	1.12 [1.07–1.18]	
	Intellectual disabilities	−0.03	0.27	−0.12	0.91	1.03 [0.6–1.77]	
	Model 2						0.035
	Age	0.03	0.01	2.4235	0.02*	1.03 [1.01–1.05]	
	Factor 1	0.21	0.04	4.8295	< 0.001**	1.23 [1.13–1.34]	
	Intellectual disabilities	0.08	0.28	0.29	0.77	1.09 [0.63–1.87]	
	Model 3						0.029
	Age	0.04	0.01	3.385	< 0.001***	1.04 [1.02–1.07]	
	Factor 2	0.17	0.04	4.048	< 0.001**	1.19 [1.09–1.29]	
	Intellectual disabilities	0.07	0.28	0.25	0.81	1.07 [0.62–1.85]	
Total current CCR	Model 1						0.023
	Age	−0.021	0.01	−2.01	0.04*	0.98 [0.97–1.00]	
	PCL:SV Total	0.01	0.02	0.932	0.35	1.01[0.98–1.04]	
	Intellectual disabilities	−0.77	0.18	−4.36	< 0.001***	0.46 [0.33–0.65]	
	Model 2						0.025
	Age	−0.021	0.01	−3.9	0.046*	0.98 [0.97–1.00]	
	Factor 1	0.045	0.03	1.4678	0.1407	1.04 [0.99–1.1]	
	Intellectual disabilities	−0.74	0.18	−4.15	< 0.001***	0.48 [0.34–0.68]	
	Model 3						0.023
	Age	−0.021	0.01	−2.04	0.046*	0.98 [0.97–1.00]	
	Factor 2	0.004	0.03	0.15	0.8863	1 [0.95–1.06]	
	Intellectual disabilities	−0.78	0.18	−4.4	< 0.001***	0.46 [0.33–0.65]	
Total violent offenses	Model 1						0.012
	Age	0	0.01	0.134	0.69	1.00 [0.98–1.02]	
	PCL:SV Total	0.05	0.02	2.4557	0.01*	1.05 [1.01–1.09]	
	Intellectual disabilities	−0.55	0.22	−2.49	0.01*	0.58 [0.37–0.89]	
	Model 2						0.011
	Age	0	0.01	0.00422	0.9982	1.00 [0.98–1.02]	
	Factor 1	0.079	0.03	2.518	0.03*	1.08 [1.01–1.15]	
	Intellectual disabilities	−0.52	0.23	−2.31	0.02*	0.59 [0.38–0.92]	
	Model 3						0.011
	Age	0.001	0.01	0.2753	0.796	1.00 [0.98–1.02]	
	Factor 2	0.07	0.03	2.03	0.04*	1.07 [1.00–1.14]	
	Intellectual disabilities	−0.6	0.22	−2.7	< 0.01**	0.55 [0.36–0.85]	

Panel (b)		*β*	SE	*z*	*p*	OR (95% CI)	Pseudo *R* ^2^ (McFaddon)
Forensic background	PCL:SV Total	0.06	0.02	2.658	0.01*	1.06 [1.02–1.11]	0.069
	Intellectual disabilities	−1.07	0.27	−3.98	< 0.001***	0.34 [0.20–0.58]	0.1
	Factor 1	0.248	0.06	3.92	< 0.001***	1.27 [1.13–1.44]	
	Factor 2	−0.11	0.06	−1.98	0.047*	0.90 [0.80–1.00]	
	Intellectual disabilities	−0.88	0.28	−3.14	< 0.01*	0.42 [0.24–0.72]	
Diagnosis of PD	PCL:SV Total	0.11	0.03	3.9285	< 0.001***	1.12 [1.06–1.18]	0.069
	Intellectual disabilities	0.43	0.33	1.33	0.18	1.54 [0.82–2.96]	
	Factor 1	0.262	0.076	3.8348	< 0.001***	1.30 [1.14–1.49]	0.093
	Factor 2	−0.04	0.07	−0.62	0.53	0.96 [0.84–1.09]	
	Intellectual disabilities	0.7	0.35	1.98	0.05	2.00 [1.02–4.07]	
Mental Health Act section	PCL:SV Total	0.04	0.02	1.6	0.109	0.96 [0.92–1.01]	0.045
	Intellectual disabilities	−0.91	0.27	−3.43	< 0.001***	0.40 [0.24–0.67]	
	Factor 1	0.19	0.06	3.3	0.00**	1.21 [1.08–1.36]	0.074
	Factor 2	−0.11	0.05	−2.04	0.04*	0.89 [0.80–0.99]	
	Intellectual disabilities	−0.74	0.28	−2.66	0.01*	0.48 [0.28–0.82]	

Panel (c)		Location	*β*	SE	*z*	*p*	OR (95% CI)	Pseudo *R* ^2^ (McFaddon)
Model 1	PCL:SV Total	Transferred	−0.01	0.03	−0.43	0.67	0.99 [0.93–1.04]	0.051
		Discharged	−0.13	0.03	−4.34	< 0.001***	0.87 [0.82–0.93]	
	Intellectual disabilities	Transferred	−0.23	0.33	−0.68	0.5	0.80 [0.42–1.53]	
		Discharged	0.38	0.32	1.18	0.24	1.46 [0.78–2.73]	
Model 2	Factor 1	Transferred	−0.13	0.07	−1.85	0.06	0.98 [0.87–1.12]	0.052
		Discharged	−0.01	0.07	−0.27	0.79	0.88 [0.77–1.01]	
	Factor 2	Transferred	−0.14	0.07	−2.12	0.03*	0.99 [0.87–1.13]	
		Discharged	−0.008	0.07	−0.12	0.9	0.87 [0.77–0.99]	
	Intellectual disabilities	Transferred	0.36	0.33	1.06	0.29	0.78 [0.40–1.53]	
		Discharged	−0.25	0.35	−0.73	0.47	1.43 [0.74–2.74]	

Panel (d)		*β*	SE	*z*	*p*	OR (95% CI)	Pseudo *R* ^2^ (McFaddon)
Changes in security ward	PCL:SV Total	0.1	0.03	3.06	< 0.01**	1.10 (1.04–1.18)	0.061
	Intellectual disabilities	−0.061	0.35	−1.75	0.08	0.55 [0.27–1.07]	
	Factor 1	0.145	0.06	2.465	0.01*	1.16 (1.03–1.30)	0.044
	Intellectual disabilities	−0.49	0.35	−1.42	0.16	0.61 [0.31–1.20]	
	Factor 2	0.187	0.06	3.1904	< 0.01**	1.20 (1.08–1.35)	0.065
	Intellectual disabilities	−0.71	0.35	−2.01	0.04*	0.49 [0.24–0.97]	

*Note*: Reference categories logistic regressions (PCL:SV and characteristics associated with psychopathy) = No forensic background, no diagnosis of PD, forensic section. Location in secure psychiatric hospital based on changes from location at baseline (no change, transferred, discharge). Reference category for logistic regression (changes in security ward) = no change. Significance level, *p < 0.05, **p < 0.01, ***p < 0.001.

Abbreviations: CCR = convictions, cautions, and reprimands, CLoS = current length of stay (days), IRR = incidence rate ratio, OR = odds ratio, PD = personality disorder, SE = standard error, SPC = secure psychiatric care.

Increasing PCL:SV Total and Factor 1 was associated within significantly increased odds of having a forensic background, while Factor 2 was associated with significantly decreased odds of having a forensic background, Table 3b. A one‐point increase in PCL:SV Total significantly increased the chances of having a forensic background by 1.06 times, or 6%, and a one‐point increase in Factor 1 significantly increased the chances of having a forensic background by 1.27 times, or 27%. However, the opposite was the case for Factor 2. With each of these models, having an intellectual disability was associated with a reduced odds of having a forensic background. Both PCL:SV Total and Factor 1 was significantly associated with increased odds of having a diagnosis of a personality disorder. A one‐point increase in PCL:SV Total and Factor 1 significantly increased the chances of having a personality disorder diagnosis by 1.12 times, or 12%, and 1.30 times, or 30%, respectively. Factor 2 did not significantly increase the odds of having a personality disorder diagnosis. Turning to consider the type of Mental Health Act section, only Factor 1 and Factor 2 were associated with the odds of being detained under a forensic or civil section, in opposite directions. A one‐point increase in Factor 1 was significantly associated with a 1.21 times (21%) greater chance of being detained under a forensic section, while Factor 2 was significantly associated with increased odds of being detained under a civil section. For both these models, having an intellectual disability was associated with increased odds of being detained under a civil section. All results remained consistent with the exception of the relationship between Factor 2 and the type of Mental Health Section, which was no longer significant, following our missing data sensitivity analysis (see Supporting Information).

### 2.3 Predictive Validity: Transfer and Discharge From Secure Wards

PCL:SV Total score was a significant predictor of ward location 12‐months later, Table 3c. These findings indicated that higher scores were associated with decreased odds of being discharged, relative to no change. Factor 2 was associated with a decreased odds of transfer to another ward, relative to no change, Table 3c.

There was a significant positive relationship between PCL:SV Total Score, Factor 1, and Factor 2 and the probability of experiencing no change or a negative change in ward security level at 12‐months, Table 3d. A one‐point increase in PCL:SV Total, Factor 1, and Factor 2 significantly increased the chances of experiencing a negative change or no change in ward security by 1.10 times (10%), 1.16, (16%), and 1.20 (20%) respectively. For the model including PCL:SV Total Score, and Factor 2, having an intellectual disability was associated with increased odds of experienced a positive change in ward security 12‐months later. All results remained consistent following missing data sensitivity analysis, with the exception that intellectual disability was not related to changes in ward security (see Supporting Information).

### 2.4 Predictive Validity

In the 12‐weeks prior to the 12‐month follow‐up, physical and verbal aggression were the most frequent types of aggressive/problematic behavior, while sexual behavior was the least frequent, Table 2. The PCL:SV (Total and Factor Scores) had an area under the curve (AUC) of less than 0.70 for all aggressive and problematic behaviors, indicating overall poor discriminatory ability (de Hond et al. 2022), Table 4. Despite this, the AUC estimates and logistic regression results were statistically significant for several specific behaviors. Factor 2 was the best predictor of aggressive or problematic behaviors, and AUC values showed that it was a significant predictor for all specified behaviors. The PCL:SV Total Score had a statistically significant AUC for all specified behaviors except physical aggression. Factor 1 significantly predicted all problematic behaviors with the exception of physical aggression and inappropriate behaviors. Some AUC results did not survive missing data analysis, with the AUC for Factor 2 and physical aggression changing from 0.59 (*p* = 0.03) to 0.57 (*p* = 0.07); however, changes were small and did not result in any other significant results becoming non‐significant. The relationship between PCL:SV Total Score, Factor 1, and Factor 2 did not remain significant for Threats of Aggression and Intimidating Behavior following missing data analyses (see Supporting Information).

**TABLE 4:** Logistic regression and AUC analysis: PCL:SV scores as predictors of aggressive/problematic behaviors at 12 months.

		*Β*	SE	*z*	*p*	OR (95% CI)	AUC (95% CI)	AUC *p*
Physical aggression	PCL:SV Total	0.03	0.03	0.94	0.35	1.03 [0.97–1.08]	0.57 [0.47–0.66]	0.08
	Intellectual disabilities	0.36	0.33	1.08	0.28	1.43 [0.75–2.76]		
	Factor 1	0	0.05	0.06	0.95	1.00 [0.91–1.11]	0.54 [0.45–0.63]	0.19
	Intellectual disabilities	0.33	0.33	0.97	0.33	1.39 [0.72–2.71]		
	Factor 2	0.1	0.05	1.93	0.05	1.10 [1.00–1.22]	0.60 [0.51–0.70]	0.01*
	Intellectual disabilities	0.35	0.33	1.04	0.3	1.42 [0.74–2.74]		
Verbal aggression	PCL:SV Total	0.12	0.03	3.97	< 0.001***	1.12 [1.06–1.19]	0.67 [0.59–0.76]	< 0.001***
	Intellectual disabilities	0.07	0.33	0.2	0.84	1.07 [0.56–2.04]		
	Factor 1	0.17	0.05	3.31	< 0.001***	1.18 [1.07–1.31]	0.64 [0.56–0.73]	< 0.01**
	Intellectual disabilities	0.2	0.33	0.6	0.55	1.22 [0.64–2.35]		
	Factor 2	0.22	0.05	4.13	< 0.001***	1.25 [1.13–1.39]	0.69 [0.61–0.77]	< 0.001***
	Intellectual disabilities	−0.11	0.33	−0.34	0.73	0.89 [0.46–1.71]		
Sexual behavior	PCL:SV Total	0.1	0.34	2.84	< 0.01**	1.10 [1.03–1.18]	0.69 [0.61–0.77]	< 0.01**
	Intellectual disabilities	−0.09	0.42	−0.216	0.83	0.91 [0.40–2.06]		
	Factor 1	0.15	0.06	2.4	0.01*	1.16 [1.03–1.30]	0.64 [0.54–0.75]	0.01*
	Intellectual disabilities	0.03	0.42	0.06	0.95	1.03 [0.44–2.36]		
	Factor 2	0.2	0.07	2.97	< 0.01**	1.21 [1.07–1.39]	0.68 [0.58–0.77]	< 0.01**
	Intellectual disabilities	−0.21	0.42	−0.5	0.61	0.81 [0.35–1.83]		
Violence towards self	PCL:SV Total	0.04	0.04	1.18	0.24	1.04 [0.97–1.12]	0.65 [0.55–0.75]	0.01*
	Intellectual disabilities	0.99	0.45	2.18	0.03*	2.69 [1.13–6.79]		
	Factor 1	0.03	0.07	0.5	0.62	1.03 [0.91–1.18]	0.63 [0.53–0.72]	0.02*
	Intellectual disabilities	0.98	0.46	2.12	0.03*	2.65 [1.10–6.81]		
	Factor 2	0.11	0.07	1.66	0.1	1.12 [0.98–1.27]	0.65 [0.55–0.76]	< 0.01**
	Intellectual disabilities	0.9	0.45	2	0.05	2.46 [1.04–6.17]		
Rule breaking	PCL:SV Total	0.07	0.03	2.44	0.02*	1.08 [1.02–1.14]	0.65 [0.57–0.74]	< 0.001*
	Intellectual disabilities	0.79	0.35	2.23	0.03*	2.2 [1.11–4.48]		
	Factor 1	0.13	0.05	2.35	0.02*	1.13 [1.02–1.26]	0.65 [0.56–0.73]	0.01*
	Intellectual disabilities	0.9	0.37	2.46	0.01*	2.46 [1.21–5.14]		
	Factor 2	0.13	0.05	2.38	0.02*	1.14 [1.03–1.27]	0.66 [0.57–0.74]	0.001*
	Intellectual disabilities	0.71	0.35	2.03	0.04*	2.04 [1.03–4.11]		
Threats of aggression	PCL:SV Total	0.06	0.03	2.01	0.04*	1.06 [1.00–1.13]	0.61 [0.51–0.71]	0.02*
	Intellectual disabilities	0.144	0.37	0.39	0.7	1.16 [0.56–2.40]		
	Factor 1	0.1	0.06	1.79	0.07	1.10 [0.99–1.23]	0.61 [0.51–0.71]	0.02*
	Intellectual disabilities	0.22	0.38	0.59	0.56	1.25 [0.59–2.65]		
	Factor 2	0.12	0.06	2.18	0.03*	1.13 [1.01–1.27]	0.62 [0.52–0.72]	0.01*
	Intellectual disabilities	0.08	0.37	0.22	0.82	1.09 [0.52–2.26]		
Intimidating behavior	PCL:SV Total	0.09	0.03	3.02	< 0.01**	1.09 [1.03–1.16]	0.65 [0.55–0.75]	< 0.01**
	Intellectual disabilities	−0.38	0.37	−1.05	0.3	0.68 [0.32–1.39]		
	Factor 1	0.12	0.05	2.24	0.01*	1.13 [1.02–1.25]	0.62 [0.51–0.73]	< 0.01**
	Intellectual disabilities	−0.3	0.37	−0.8	0.43	0.74 [0.35–1.53]		
	Factor 2	0.19	0.06	3.3	< 0.001***	1.21 [1.08–1.36]	0.67 [0.57–0.77]	< 0.001***
	Intellectual disabilities	−0.52	0.37	−1.39	0.16	0.60 [0.28–1.22]		
Inappropriate behavior	PCL:SV Total	0.06	0.04	1.6	0.11	1.06 [0.99–1.14]	0.62 [0.49–0.75]	0.02*
	Intellectual disabilities	0.61	0.43	1.42	0.16	1.83 [0.80–4.32]		
	Factor 1	0.06	0.06	0.89	0.37	1.06 [0.93–1.20]	0.58 [0.45–0.71]	0.09
	Intellectual disabilities	0.61	0.43	1.41	0.16	1.85 [0.79–4.42]		
	Factor 2	0.16	0.07	2.4	0.02*	1.17 [1.03–1.35]	0.67 [0.54–0.79]	0.01*
	Intellectual disabilities	0.6	0.44	1.38	0.17	1.82 [0.78–4.37]		
Overall presence	PCL:SV Total	0.09	0.03	2.76	< 0.01*	1.09 [1.03–1.16]	0.65 [0.56–0.74]	< 0.01**
	Intellectual disabilities	0.51	0.36	1.42	0.16	1.66 [0.83–3.40]		
	Factor 1	0.11	0.06	2.02	0.04*	1.12 [1.01–1.25]	0.62 [0.53–0.72]	< 0.01*
	Intellectual disabilities	0.57	0.36	1.58	0.12	1.77 [0.88–3.66]		
	Factor 2	0.19	0.06	3.19	< 0.01**	1.20 [1.08–1.36]	0.67 [0.58–0.76]	< 0.001***
	Intellectual disabilities	0.39	0.36	1.07	0.28	1.47 [0.73–3.01]		
Violent intent	PCL:SV Total	0.09	0.03	3.02	< 0.01**	1.09 [1.03–1.15]	0.66 [0.57–0.74]	< 0.001***
	Intellectual disabilities	0.58	0.33	1.78	0.08	1.78 [0.95–3.42]		
	Factor 1	0.09	0.05	1.89	0.06	1.10 [1.00–1.21]	0.61 [0.53–0.70]	< 0.01*
	Intellectual disabilities	0.6	0.33	1.84	0.07	1.83 [0.97–3.52]		
	Factor 2	0.2	0.05	3.79	< 0.001***	1.22 [1.11–1.36]	0.61 [0.61–0.77]	< 0.001***
	Intellectual disabilities	0.47	0.33	1.43	0.15	1.60 [0.84–3.08]		

*Note*: Reference category = behavior not present. Significance level, *p < 0.05, **p < 0.01, ***p < 0.001.

Abbreviations: CI = confidence intervals, SE = standard error.

### 2.5 Convergent Validity

There was a moderate degree of correlation between PCL:SV Total and HCR‐20 Total Scores, with weaker correlations between Factor 1 and Factor 2, and HCR‐20 Total Scores, Table 5. All correlations between PCL:SV Total and Factor Scores and Total and subscale scores on the HCR‐20 were positive and significant, *p* < 0.001, except for the relationship between PCL:SV Total and the Imminent Violence subscale, *r* = 0.19, *p* < 0.01, as well as Factor 1 and the Imminent Violence subscale, *r* = 0.14, *p* = 0.02. Correlations between the PCL:SV and the START risk assessment revealed a moderate degree of correlation between the PCL:SV Total and Factor Scores and the Vulnerabilities subscale only, which were all significant, *p* < 0.001. No significant relationships between the PCL:SV Total or Factor Scores and the START Strengths subscale were observed. However, when performing missing data analysis, significant negative relationships between PCL:SV Total and Factor 2, and the START Strength subscale were observed, as would be expected.

**TABLE 5:** Correlations between PCL:SV Total and Factor scores and HCR20 and START measures of clinical risk.

risk assessment tool	PCL:SV Total	PCL:SV Factor 1	PCL:SV Factor 2
HCR20			
Historical scale	*r* = 0.37, *p* < 0.001***	*r* = 0.37, *p* < 0.001***	*r* = 0.35, *p* < 0.001***
Clinical scale	*r* = 0.41, *p* < 0.001***	*r* = 0.35, *p* < 0.001***	*r* = 0.41, *p* < 0.001***
Risk management scale	*r* = 0.38, *p* < 0.001***	*r* = 0.35, *p* < 0.001***	*r* = 0.37, *p* < 0.001***
Total score	*r* = 0.40, *p* < 0.001***	*r* = 0.36, *p* < 0.001***	*r* = 0.32, *p* < 0.001***
Serious physical harm	*r* = 0.27, *p* < 0.001***	*r* = 0.21, *p* < 0.001***	*r* = 0.30, *p* < 0.001***
Imminent violence	*r* = 0.19, *p* < 0.01**	*r* = 0.14, *p* = 0.02*	*r* = 0.22, *p* < 0.001***
Future violence	*r* = 0.30, *p* < 0.001***	*r* = 0.24, *p* < 0.001***	*r* = 0.31, *p* < 0.001***
START			
Strengths	*r* = −0.01, *p* = 0.83	*r* = 0.06, *p* = 0.35	*r* = −0.06, *p* = 0.34
Vulnerabilities	*r* = 0.45, *p* < 0.001***	*r* = 0.32, *p* < 0.001***	*r* = 0.50, *p* < 0.001***

*Note*: Significance level, *p < 0.05, **p < 0.01, ***p < 0.001.

## 3 Discussion

Instruments like the PCL:SV are often used in populations that differ from those in which the measure was developed. This can be problematic, leading to invalid assessments and is particularly problematic within the CJS where labeling an individual as a psychopath can influence decision‐making and foster punitive actions (Blais and Forth 2014). Within this study, we aimed to explore the factor structure, reliability, and validity of the PCL:SV in autistic adults within psychiatric hospitals, a majority of whom were within forensic psychiatric hospitals. Their mean PCL:SV Total Score was similar to or slightly lower than that reported within studies that included forensic inpatients without autism (Bo et al. 2019; Ho et al. 2009; Pedersen et al. 2010), but higher than that reported within studies using non‐forensic non‐autistic inpatients (Doyle et al. 2002; Nicholls et al. 2004), including those within the MacArthur Violence Risk Assessment Study (Skeem and Mulvey 2001).

Turning to consider our factor analysis, we were able to fit a two‐factor model that was similar to that originally reported for the PCL:SV (Hart et al. 1995). This model was chosen due to its similarity to the original two‐factor PCL:SV structure and the associated scoring as found within the published manual. However, we included residual covariance between some variables within each factor to ensure a good fit. It is worth noting that there is some debate within the literature as to whether psychopathy is best represented by a two‐ or three‐factor model. For example, Cooke and Michie (2001) proposed a three‐factor model of psychopathy using PCL‐R data incorporating: (a) arrogant and deceitful interpersonal style (glibness, superficial charm, grandiosity, pathologically lying, and conning/manipulative), (b) deficient affective experience (shallow affect and callousness, lack of empathy, lack of remorse, and failure to accept responsibility), and (c) impulsive and irresponsible behavior style (need for stimulation/boredom, impulsivity, irresponsibility, parasitic lifestyle, and lack of realistic goals). While our goal was not to test the validity of a two‐factor versus a three‐factor model of psychopathy using PCL:SV data, as there continues to be much debate, it is important to note that others have reported that a three‐factor PCL:SV model is superior to a two‐factor model using data captured from forensic psychiatric inpatients (Bo et al. 2019). While there is support for a three‐factor PCL:SV relative to a unitary, two‐factor, and four‐factor model in some studies (Veal et al. 2021), this has not been consistent and a single, two‐, three‐, or four‐factor model has been reported. Boduszek et al. (2016) reported a bifactor PCL:SV model with two‐factors was superior to nine alternative PCL:SV models. It would be appropriate to undertake a future study investigating PCL:SV measurement invariance within autistic people and alternative and potentially better fitting factor structures.

Further, we found that the PCL:SV had good to excellent reliability and construct validity, particularly the PCL:SV Total and Factor 1 scores. Factor 2 showed a more intricate relationship with psychopathy‐related traits, with some relationships reversed. Regarding predictive validity, higher PCL:SV scores were associated with factors indicating poorer treatment progress. Predictive validity for aggressive or problematic behaviors was limited. However, Factor 2 predicted aggressive and problematic behaviors more robustly, potentially influenced by observable behavior associated with autism and/or intellectual disabilities (e.g., behaviors that challenge). Generally, the results aligned with existing research and theoretical understanding of psychopathy and the PCL:SV.

### 3.1 Reliability

Reliability analyses revealed robust estimates of internal consistency for the PCL:SV, with McDonald's Omega indicating excellent structural reliability. Reliability estimates were lowest for Factor 2, similar to prior research in different populations (Hart et al. 1995; Rogers et al. 2000; Žukauskienė et al. 2010). This may represent potential variability across populations, with different groups of people exhibiting a wider variation in the lifestyle/behavioral characteristics measured by Factor 2. Alternatively, this could be due to increased difficulty in measuring these behavioral characteristics.

### 3.2 Construct Validity

Positive associations between the PCL:SV and variables that are thought to be associated with psychopathy were found, including length of stay, admissions, previous convictions, and the number of violent offenses. Factor 1 was associated with an increased likelihood of having a forensic background, more violent offenses, and detention under a forensic section of the Mental Health Act, 1983. Factor 2 was also associated with having more violent offenses. This aligns with previous research on psychopathy, which has long been associated with criminal and violent behavior (Dhingra and Boduszek 2013; Hare 1999), longer offending trajectories, and an increased risk of re‐institutionalization after release from prison (Porter et al. 2001).

PCL:SV Total and Factor 1 scores were significantly and positively related to having a personality disorder. Antisocial personality disorder (ASPD) is considered the closest diagnostic category to psychopathy (Ogloff 2006). Indeed, some research suggests that psychopathy is at the extreme end of ASPD (Coid and Ullrich 2010), with most psychopaths also meeting ASPD criteria (Hare 1996). No relationship was observed between Factor 2 and personality disorder. Factor 1 encompasses “core” personality features of psychopathy, capturing characteristics such as superficiality, deceitfulness, and lack of empathy, which may be more closely related to the central features of personality disorders than Factor 2 characteristics (Swales 2022).

In contrast to Factor 1, Factor 2 was associated with a greater likelihood of being detained under a civil section of the Mental Health Act, 1983, and a reduced likelihood of having a forensic background. In line with this, a systematic review by Collins et al. (2022) reported that autistic people who encounter the CJS are less likely to have a forensic history, with fewer previous convictions than non‐autistic offenders. Consideration of the specific behavioral characteristics captured by Factor 2, such as impulsivity and poor behavioral control, this is unsurprising. Those presenting with behaviors that challenge, as seen more commonly amongst those with intellectual disabilities, may be more likely to score on Factor 2. It is worth noting that a substantial proportion (49%) of our participant population had an intellectual disability. Our findings across our models indicated that autistic inpatients with intellectual disabilities were less likely to have current convictions, cautions or reprimands, violent offenses, and a forensic background. They were more likely to be detained under a civil section of the Mental Health Act, and more likely to have had a positive transfer to a ward of lesser security 12‐months later. Accurate levels of antisocial behavior may be hard to capture in adults with intellectual disability (Morrissey, Hogue, et al. 2007); carers often show a reluctance to report offending behaviors to the police (McBrien and Murphy 2006) and once reported, individuals are less likely to be charged for antisocial acts (Cockram 2005). It is also noteworthy that approximately 28% of autistic people have comorbid attention‐deficit hyperactivity disorder (ADHD; Lai et al. 2019), which is characterized by inattention, hyperactivity, and impulsivity (World Health Organization 2021). Co‐morbid ADHD could further contribute towards high scores on Factor 2. It has previously been reported that although the emotional features of psychopathy are not impaired in ADHD, the behavioral features of psychopathy are present (Eisenbarth et al. 2008), suggesting behavioral overlap between Factor 2 and ADHD.

No relationship was found between the PCL:SV and current convictions, cautions or reprimands, which may be due to two reasons, recognizing that those with intellectual disabilities were less likely to have current convictions, cautions or reprimands. First, most participants had current convictions, cautions or reprimands. However, we only had frequency data and not severity data. It may be expected that a proportion of those with higher PCL:SV scores would commit more severe, but less frequent offenses (e.g., murder) relative to those with lower PCL:SV scores. Second, detained individuals have restricted movement, increased supervision, and risk management plans; actions which collectively reduce opportunities to engage in offending behaviors while in hospital.

### 3.3 Predictive Validity

PCL:SV Total score was significantly associated with decreased likelihood of being discharged, while Factor 2 was associated with decreased likelihood of transfer. PCL:SV Total and Factor Scores were also associated with increased likelihood of experiencing negative change/no change in security level at 12 months. This is unsurprising given that psychopathic traits are predictive of poor therapeutic progress and risk reduction (Olver et al. 2013), both of which are critical considerations when applying to transfer to a lower security ward (NHS England 2021). Research on the predictive validity of the PCL:SV and treatment progress is sparse. However, similar results have been reported with regards to the predictive validity of the PCL‐R in offenders with intellectual disability (Morrissey, Mooney, et al. 2007) and in individuals with personality disorders detained in high‐security settings (Tetley et al. 2010). In this study, Factor 1 and Factor 2 were both significant predictors of negative/no change in security level, indicating that both interpersonal and lifestyle factors can inhibit treatment progress. This contrasts to the existing PCL‐R research where Factor 1 is reported as a stronger predictor of negative treatment progression (Morrissey, Mooney, et al. 2007; Olver et al. 2013; Tetley et al. 2010). Interpersonal factors such as superficiality and lack of remorse, likely hinder therapeutic rapport building and insight development, resulting in reduced opportunities for treatment progress (Olver et al. 2013).

Differences in study design may contribute to varied findings regarding the influence of Factor 2. However, it is also necessary to consider the specific needs and behaviors of autistic adults within psychiatric hospitals. Not all patients in secure settings have a forensic background; some are admitted due to behaviors that challenge, which cannot be safely managed within general psychiatric services (Völlm et al. 2018), while not all our participants were detained with forensic psychiatric wards. Behaviors that challenge are common in autistic individuals and include behaviors such as aggression, destructive behavior, and self‐injurious or stereotyped behavior (Matson and Rivet 2008). Managing these behaviors can be complex, posing challenges in maintaining a safe environment (Matson et al. 2011). Thus, behaviors that challenge may be driving Factor 2 as a stronger predictor of negative/no change in security level within the current sample.

The predictive validity of the PCL‐SV for problematic or aggressive behaviors was explored but was limited. Nonetheless, Factor 2 was associated with significant AUC values for several behaviors and was the more robust predictor, relative to Factor 1, and PCL‐SV Total Scores. Again, this may be influenced by the specific behavioral problems exhibited by autistic individuals. As the study looked at the presence versus absence of behaviors, rather than intensity or frequency, it is possible that individuals scoring highly on Factor 2 exhibited high frequency but low intensity behaviors (e.g., shouting and banging) rather than low frequency but higher intensity behaviors (e.g., sexual assault or violent attacks) which may be more associated with psychopathy than autism. Broadening the sample to encompass autistic individuals across different settings may enhance the predictive validity of the PCL:SV as the current sample is biased towards those prone to display a greater frequency of problematic or aggressive behaviors.

### 3.4 Convergent Validity

The PCL:SV demonstrated convergent validity with other risk assessment tools. Significant and moderate correlations were observed between the PCL:SV, HCR‐20, and START, except for the START strengths scale. Overall, the measures were expected to align but not be highly correlated as they focus on the measurement of different constructs or risk factors. However, it is important to note that there is a sparsity of research validating the HCR‐20 and the START as risk assessment tools for use with autistic adults.

### 3.5 Clinical Implications

Overall, these findings suggest that the PCL:SV has good reliability and validity when used with autistic adults within psychiatric hospitals. This allows for a more robust assessment of risk, as well as a more effective investigation of the “double hit” hypothesis (Rogers et al. 2006) in future studies. Individuals with both autism and psychopathy represent a small but clinically significant subgroup of autistic offenders with unique treatment needs (Alexander et al. 2016). Barnoux et al. (2020) suggested that autistic individuals with a comorbid diagnosis of psychopathy present with increased forensic risk, requiring longer stays in secure psychiatric care, while those without a diagnosis of psychopathy may benefit from shorter stays and community‐based placements. Validated psychometric tools such as the PCL:SV can aid in the accurate assessment and diagnosis of psychopathy in autistic individuals, ultimately enhancing treatment outcomes and risk management.

### 3.6 Strengths and Limitations

The relatively large dataset from a range of inpatient services enhanced the generalizability of the study findings. The confirmatory factor analysis was consistent with the results derived from participants without autism, and missing data imputation showed that the data were largely unbiased, strengthening the integrity of the findings. While the intelligence quotient was unavailable for all participants, we were able to include whether participants had a diagnosis of an intellectual disability within our analysis. Considering limitations, data on aggressive/problematic behavior indexed the frequency of behaviors and not severity. Failure to account for this distinction complicates the differentiation between high‐frequency, low‐intensity behaviors potentially associated with autism rather than psychopathy (e.g., behaviors that challenge) and may contribute to the reduced predictive validity of the PCL:SV. Further, and arguably, it is possible that some autistic individuals may score on the PCL:SV due to the nature or degree of their autism. We were unable to investigate this possibility explicitly within our study because we did not capture data about the degree of autistic symptomatology. However, as PCL:SV Factor 1 captures difficulties with empathy, it could be argued that Factor 1 should overlap with autism and therefore predict violence poorly, relative to Factor 2. This was not the case as both Factors predicted history of violent offending and future aggressive and problematic behavior. However, Factor 1 was associated with having a forensic background, a diagnosis of personality disorder, and detention under a forensic section of the Mental Health Act, while the reverse was the case for Factor 2. Further, and while it is highly unlikely that those who completed the PCL:SV also diagnosed our participants with autism, we cannot guarantee this to be the case in all instances as we did not capture these data. Finally, the PCL:SV was used by clinicians within the intended setting, but we did not undertake reliability checks, which may have introduced some error; nevertheless, our findings indicated that the PCL:SV is an appropriate measure for use with autistic psychiatric inpatients.

### 3.7 Constraints Upon Generality

There is evidence about the validity, reliability, and factor structure of the PCL‐SV when used with community and psychiatric participants drawn from various countries (Veal et al. 2021), but no robust evidence supporting the use of the PCL‐SV with autistic psychiatric inpatients. Within this study, we aimed to evaluate the performance of the PCL‐SV when used with autistic psychiatric inpatients in England and Wales. A majority were detained in secure psychiatric services, and some had intellectual disabilities, while the majority were men; we sampled from numerous hospitals helping to ensure that our sample is representative at two time points separated by 12‐months. Researchers would need to sample in a similar way over a similar time period to generate comparable results. Our findings are limited to autistic psychiatric inpatients in England and Wales as we did not include autistic psychiatric inpatients from other countries, nor did we include autistic people from the community. Further, we did not include autistic prisoners. Our findings do not generalize to these additional autistic populations. We have no reason to believe that the results depend on other characteristics of the participants, materials, or context.

### 3.8 Future Research

The findings from this study indicated that the PCL‐SV can be used effectively with autistic psychiatric inpatients. Future studies should attempt to disentangle the relationship between autism and psychopathy further.

## Ethics Statement

The authors assert that all procedures contributing to this work comply with the ethical standards of the relevant national and institutional committees on human experimentation and with the Helsinki Declaration of 1975, as revised in 2008.

## Conflicts of Interest

The authors declare no conflicts of interest.

## Supporting information


Data S1.

## Data Availability

The data that support the findings of this study are available on request from the corresponding author. The data are not publicly available due to privacy or ethical restrictions.
