# Supplementary material for: The Factor Structure and Validity of the Psychopathy Checklist‐Short Version When Used With Autistic Psychiatric Inpatients
Source: Autism Res. 2025 Feb 18;18(3):614–31. doi: 10.1002/aur.70004 (PMC11928917; doi:10.1002/aur.70004)
Supplement: Supplementary file 1 — Data S1. [file AUR-18-614-s001.docx]

**Supplemental Materials: Tables 6 to 9**

| **Table 6**  *Descriptive statistics for included variables grouped by level of psychopathy based upon PCL:SV scores* | | | | | | | | | | | | |
| --- | --- | --- | --- | --- | --- | --- | --- | --- | --- | --- | --- | --- |
|  | *Not Psychopathic (n = 165)* | | | *Maybe Psychopathic (n =53)* | | | | *Likely Psychopathic (n = 38)* | | | | *Post hoc Testing^1^* |
|  | *n =* | *M =* | *SD =* | | n = | *M =* | *SD =* | | n = | M = | *SD =* |  |
| PCL:SV Total | 165 | 7.19 | 3.42 | | 53 | 14.66 | 1.43 | | 38 | 20.47 | 2.13 | NP<MP*; NP<LP*; MP<LP* |
| Factor 1 | 165 | 3.10 | 2.02 | | 53 | 6.25 | 2.00 | | 38 | 10.13 | 1.79 | NP<MP*; NP<LP*; MP<LP* |
| Factor 2 | 165 | 4.03 | 2.28 | | 53 | 8.42 | 1.65 | | 38 | 10.42 | 1.55 | NP<MP*; NP<LP*; MP<LP* |
| CLoS | 165 | 724.07 | 829.15 | | 53 | 969.85 | 1450.61 | | 38 | 1193.24 | 1353.28 | NP<MP; NP<LP*; MP<LP |
| START – Vulnerabilities | 160 | 19.43 | 6.95 | | 51 | 24.14 | 7.53 | | 37 | 25.97 | 6.86 | NP<MP*; NP<LP*; MP<LP |
| START – Strengths | 160 | 17.64 | 7.65 | | 51 | 14.76 | 7.48 | | 37 | 17.92 | 8.29 | NP<MP*; NP<LP*; MP<LP |
| HCR-20 Total Risk | 160 | 25.98 | 7.49 | | 51 | 29.84 | 6.11 | | 37 | 32.28 | 5.17 | NP<MP; NP<LP*; MP<LP |
| Total Days Spent in SPC | 162 | 2292.93 | 2911.61 | | 51 | 3731.12 | 4305.99 | | 37 | 2954.54 | 2855.54 | NP<MP*; NP<LP*; MP<LP |
| Total Previous CCR | 158 | 2.45 | 5.51 | | 52 | 5.98 | 16.75 | | 36 | 12.39 | 39.43 | NP<MP; NP<LP*; MP<LP |
| Total Current CCR | 164 | 1.88 | 3.51 | | 52 | 1.71 | 2.62 | | 38 | 2.03 | 1.98 | NP<MP; NP<LP; MP<LP |
| Total Violent Offences | 145 | 2.28 | 4.66 | | 47 | 2.94 | 5.34 | | 36 | 2.92 | 301 | NP<MP; NP<LP; MP<LP |
| Physical Aggression | 105 | 1.64 | 6.01 | | 31 | 1.29 | 3.22 | | 33 | 1.30 | 4.00 | NP<MP; NP<LP; MP<LP |
| Verbal Aggression | 105 | 1.01 | 2.81 | | 31 | 1.45 | 2.19 | | 33 | 3.30 | 4.26 | NP<MP; NP<LP*; MP<LP* |
| Sexual Behaviour | 105 | .38 | 1.35 | | 31 | .45 | 1.31 | | 33 | .51 | .87 | NP<MP; NP<LP; MP<LP |
| Violence To Self | 105 | .32 | 1.36 | | 31 | 1.58 | 6.84 | | 33 | .64 | 2.44 | NP<MP; NP<LP; MP<LP |
| Rule Breaking | 105 | .49 | 1.59 | | 31 | .90 | 2.53 | | 33 | 1.15 | 2.74 | NP<MP; NP<LP; MP<LP |
| Threats of Violence/ Aggression | 105 | .47 | 1.54 | | 31 | 1.06 | 3.26 | | 33 | 1.33 | 3.63 | NP<MP; NP<LP; MP<LP |
| Intimidating Behaviour | 105 | .50 | 1.73 | | 31 | 1.45 | 4.14 | | 33 | 1.97 | 4.03 | NP<MP; NP<LP*; MP<LP |
| Inappropriate Behaviour | 105 | .65 | 3.07 | | 31 | 1.58 | 5.84 | | 33 | 1.00 | 2.50 | NP<MP; NP<LP; MP<LP |
| Total Frequency | 105 | 5.46 | 10.14 | | 31 | 9.71 | 17.49 | | 33 | 11.21 | 15.92 | NP<MP; NP<LP; MP<LP |
|  |  | % |  | |  | % |  | |  | % |  |  |
| Violent Intent | 50 | 47.6^2^ |  | | 18 | 56.3^2^ |  | | 26 | 78.8^2^ |  | <NP*; MP; >LP* |
| Forensic Background | 94 | 57.3^2^ |  | | 30 | 56.62^2^ |  | | 30 | 78.92^2^ |  | NP; MP;>LP* |
| Diagnosis of Personality Disorder | 24 | 14.5^2^ |  | | 11 | 20.8^2^ |  | | 19 | 42.1^2^ |  | <NP*, MP, >LP* |
| Diagnosis of Intellectual Disability | 81 | 49.1^2^ |  | | 28 | 52.8^2^ |  | | 15 | 39.5^2^ |  | NP; MP; LP |
| Forensic Mental Health Section | 82 | 53.6^2^ |  | | 26 | 49.1^2^ |  | | 26 | 70.3^2^ |  | NP; MP; LP |
| *Note: NP = not psychopathic, MP = maybe psychopathic, LP = likely psychopathic, CLoS = current length of stay (days), SPC = secure psychiatric care, CCR = convictions, cautions and reprimands, *p < .05. ^1^Non-parametric bootstrapping with 5000 resamples with replacement was used to make post hoc comparisons with an adjustment to the familywise error rate. ^2^Percentage of participants within the category rather than the total; for post hoc testing, comparison is made with the expected count.* | | | | | | | | | | | | |

| **Table 7**  *Percentage of missing data in variables* | |  |
| --- | --- | --- |
| Variable | Missing Data (%) |  |
| PCL:SV Total | 9 |  |
| PCL:SV Factor 1 | 9 |  |
| PCL:SV Factor 2 | 9 |  |
| Age | 0 |  |
| Current Length of Stay (days) | 0 |  |
| Total Days Spent in Mental Health Hospitals | 4 |  |
| Total Previous Convictions, Cautions and Reprimands | 4 |  |
| Total Current Convictions, Cautions and Reprimands | 1 |  |
| Total Number of Violent Offences | 11 |  |
| Forensic Background | 0 |  |
| Diagnosis of Personality Disorder | 0 |  |
| Mental Health Section | 0 |  |
| Location (at 12 month follow up) | 0 |  |
| Change in Security Level | 20 |  |
| Physical Aggression | 38 |  |
| Verbal Aggression | 38 |  |
| Sexual Behaviour | 38 |  |
| Violence Towards Self | 38 |  |
| Rule Breaking | 38 |  |
| Threats of Aggression | 38 |  |
| Intimidating Behaviour | 38 |  |
| Inappropriate Behaviour | 38 |  |
| Overall Presence | 38 |  |
| Violent Intent | 37 |  |
| HCR20 - Historical Scale | 37 |  |
| HCR20 - Clinical Scale | 7 |  |
| HCR20 - Risk Management Scale | 7 |  |
| HCR20 - Total Score | 7 |  |
| HCR20 - Serious Physical Harm | 15 |  |
| HCR20 - Imminent Violence | 16 |  |
| HCR20 - Future Violence | 15 |  |
| START - Strengths | 7 |  |
| START – Vulnerabilities  Diagnosis of LD | 7  0 |  |

| **Table 8** | | | | | | |  |  |
| --- | --- | --- | --- | --- | --- | --- | --- | --- |
| *(a) Missing data analysis, Negative binomial regression: The relationship between PCL:SV Total and Factor scores and length of stay and criminal offending history.* | | | | | | |  |  |
|  |  | β | SE | z | *p* | IRR (95% CI) | Pseudo R^2^ (McFaddon) |  |
| CLoS | Model 1 |  |  |  |  |  | 0.0051 |  |
|  |  |  |  |  |  |  |  |  |
|  |  |  |  |  |  |  |  |  |
|  | Age | 0.03 | 0.01 | 5.13 | <.01*** | 1.03 [1.01 – 1.04] |  |  |
|  | PCL:SV Total | 0.03 | 0.01 | 1.98 | <.001** | 1.03 [1.02 - 1.06] |  |  |
|  | Intellectual Disabilities | -0.05 | 0.13 | -0.38 | 0.07 | .95 [.74 – 1.23] |  |  |
|  | Model 2 | | | | | | 0.0041 |  |
|  | Age | 0.03 | 0.01 | 5.09 | <.001*** | 1.02 [1.01 – 1.03] |  |  |
|  |  |  |  |  |  |  |  |  |
|  | Factor 1 | 0.05 | 0.02 | 1.98 | <.01** | 1.06 [1.02 – 1.11] |  |  |
|  | Intellectual Disabilities | -0.012 | 0.14 | -0.09 | 0.9 | .99 [0.76 – 1.29] |  |  |
|  |  |  |  |  |  |  |  |  |
|  | Model 3 | | | | | | 0.0053 |  |
|  | Age | 0.03 | 0.01 | 4.96 | <.01** | 1.02 [1.00 – 1.03] |  |  |
|  |  |  |  |  |  |  |  |  |
|  | Factor 2 | 0.04 | 0.02 | 1.82 | <.001*** | 1.07 [1.03 -1.12] |  |  |
|  | Intellectual Disabilities | -0.07 | 0.13 | -0.56 | 0.58 | .91 [.72 – 1.21] |  |  |
| Total Days Spent in SPC | Model 1 | | | | | | 0.022 |  |
|  | Age | 0.05 | 0.01 | 9.4 | <.001*** | 1.05 [1.04 – 1.07] |  |  |
|  | PCL:SV Total | 0.03 | 0.01 | 3.01 | <.001*** | 1.03 [1.04 - 1.06] |  |  |
|  | Intellectual Disabilities | -0.07 | 0.13 | -0.56 | 0.58 | .93 [0.73 – 1.19] |  |  |
|  | Model 2 |  |  |  |  |  | 0.022 |  |
|  | Age | 0.05 | 0.01 | 9.52 | <.001*** | 1.06 [1.05 – 1.07] |  |  |
|  | Factor 1 | 0.07 | 0.02 | 2.65 | <.001*** | 1.07 [1.03 – 1.12] |  |  |
|  | Intellectual Disabilities | -0.03 | 0.12 | -0.25 | 0.8 | 0.97 [0.75 – 1.24] |  |  |
|  | Model 3 |  |  |  |  |  | 0.022 |  |
|  | Age | 0.05 | 0.01 | 9.3 | <.001*** | 1.05 [1.04 – 1.06] |  |  |
|  | Factor 2 | 0.05 | 0.02 | 2.27 | <.01** | 1.05 [1.01 – 1.9] |  |  |
|  | Intellectual Disabilities | -0.11 | 0.13 | -0.83 | 0.41 | .90 [0.71 – 1.16] |  |  |
| Total Previous CCR | Model 1 |  |  |  |  |  | 0.036 |  |
|  | Age | 0.01 | 0.01 | 1.06 | <.01** | 1.01 [.99 – 1.04] |  |  |
|  | PCL:SV Total | 0.2 | 0.05 | 4 | <.001*** | 1.22 [1.10 - 1.34] |  |  |
|  | Intellectual Disabilities | 0.02 | 0.3 | 0.08 | 0.94 | 1.02 [0.56 – 1.88] |  |  |
|  | Model 2 |  |  |  |  |  | 0.035 |  |
|  | Age | 0.02 | 0.01 | 1.94 | 0.06 | 1.03 [1.00 – 1.05] |  |  |
|  | Factor 1 | 0.21 | 0.04 | 4.8295 | <.01** | 1.17 [1.06 – 1.29] |  |  |
|  | Intellectual Disabilities | 0.1 | 0.3 | 0.33 | 0.74 | 1.10 [0.61 – 1.99] |  |  |
|  | Model 3 |  |  |  |  |  | 0.029 |  |
|  |  |  |  |  |  |  |  |  |
|  | Age | 0.02 | 0.01 | 1.94 | <.001*** | 1.03 [1.00 – 1.05] |  |  |
|  |  |  |  |  |  |  |  |  |
|  |  |  |  |  |  |  |  |  |
|  | Factor 2 | 0.16 | 0.05 | 3.15 | <.001** | 1.17 [1.06 – 1.29] |  |  |
|  | Intellectual Disabilities | 0.1 | 0.3 | 0.33 | 0.74 | 1.10 [0.62 – 1.99] |  |  |
| Total Current CCR | Model 1 |  |  |  |  |  | 0.023 |  |
|  | Age | -0.02 | 0.01 | -2.6 | <.01* | .98 [.97 – 1.00] |  |  |
|  | PCL:SV Total | 0.01 | 0.02 | 0.4 | 0.69 | 1.01[.98 – 1.04] |  |  |
|  | Intellectual Disabilities | -0.79 | 0.17 | -4.56 | <.001*** | .46 [.33 – .64] |  |  |
|  |  |  |  |  |  |  |  |  |
|  | Model 2 |  |  |  |  |  | 0.025 |  |
|  | Age | -0.02 | 0.01 | -2.64 | <0.01** | .98 [.97 – .99] |  |  |
|  | Factor 1 | 0.02 | 0.03 | 0.88 | 0.38 | 1.02 [0.97– 1.08] |  |  |
|  | Intellectual Disabilities | -0.78 | 0.18 | -4.45 | <.001*** | .46 [.33 – .65] |  |  |
|  |  |  |  |  |  |  |  |  |
|  | Model 3 |  |  |  |  |  | 0.023 |  |
|  | Age | -0.02 | 0.01 | -2.58 | 0.01* | .98 [.97 – 1.00] |  |  |
|  | Factor 2 | -0.01 | 0.03 | -0.3 | 0.76 | .99 [.94 – 1.04] |  |  |
|  | Intellectual Disabilities | -0.78 | 0.18 | -4.5 | <.001*** | .46 [.33 – .65] |  |  |
|  |  |  |  |  |  |  |  |  |
| Total Violent Offences | Model 1 |  |  |  |  |  | 0.012 |  |
|  | Age | 0 | 0.01 | -0.05 | 0.96 | 1.00 [.98 – 1.02] |  |  |
|  | PCL:SV Total | 0.05 | 0.02 | 2.63 | .01* | 1.05 [1.01 – 1.09] |  |  |
|  | Intellectual Disabilities | -0.53 | 0.21 | -2.61 | .01* | .59 [.39 – .88] |  |  |
|  | Model 2 |  |  |  |  |  | 0.011 |  |
|  | Age | 0 | 0.01 | -0.14 | 0.89 | 1.00 [.98 – 1.02] |  |  |
|  | Factor 1 | 0.08 | 0.03 | 2.42 | .02* | 1.08 [1.01 – 1.16] |  |  |
|  | Intellectual Disabilities | -0.52 | 0.23 | -2.31 | .02* | .60 [.40 – .90] |  |  |
|  | Model 3 |  |  |  |  |  | 0.011 |  |
|  | Age | 0 | 0.01 | 0.09 | 0.93 | 1.00 [.98 – 1.02] |  |  |
|  | Factor 2 | 0.06 | 0.03 | 1.96 | 0.06 | 1.07 [1.00 – 1.14] |  |  |
|  | Intellectual Disabilities | -0.56 | 0.21 | -2.7 | <.01** | .57 [.38 – .86] |  |  |

| *(b) Missing data analysis, Logistic regression: PCL:SV and forensic background, personality disorder, and Mental Health Act section* | | | | | | |  |  |
| --- | --- | --- | --- | --- | --- | --- | --- | --- |
|  |  | β | SE | z | *p* | OR (95% CI) | Pseudo R^2^ (McFaddon) |  |
| Forensic Background | PCL:SV Total | 0.06 | 0.02 | 2.52 | .01* | 1.06 [1.01 – 1.11] | 0.069 |  |
|  | Intellectual Disabilities | -1.05 | 0.26 | -4.1 | <.001*** | .35 [.2 – .58] |  |  |
|  |  |  |  |  |  |  |  |  |
|  |  |  |  |  |  |  | 0.1 |  |
|  | Factor 1 | 0.18 | 0.06 | 3.28 | <.001*** | 1.20 [1.07 – 1.33] |  |  |
|  | Factor 2 | -0.06 | 0.05 | -1.22 | .047* | .94 [.857 – 1.04] |  |  |
|  | Intellectual Disabilities | -0.9 | 0.26 | -3.41 | <.001*** | .41 [.24 – .68] |  |  |
| Diagnosis of PD | PCL:SV Total | 0.12 | 0.03 | 4.07 | <.001*** | 1.12 [1.06 – 1.18] | 0.069 |  |
|  | Intellectual Disabilities | 0.45 | 0.32 | 1.43 | 0.15 | 1.57 [.84 – 2,94] |  |  |
|  | Factor 1 | 0.24 | 0.07 | 3.56 | <.001*** | 1.26 [1.11 – 1.44] | 0.093 |  |
|  | Factor 2 | -0.01 | 0.06 | -0.13 | 0.89 | .99 [.88 – 1.12] |  |  |
|  |  |  |  |  |  |  |  |  |
|  | Intellectual Disabilities | 0.65 | 0.34 | 1.91 | 0.06 | 1.91 [.98 - 3.72] |  |  |
| Mental Health Act Section | PCL:SV Total | 0.04 | 0.02 | 1.7 | 0.09 | 1.04 [.99 – 1.09] | 0.045 |  |
|  | LD | -0.88 | 0.25 | -3.46 | <.001*** | .42 [.25 - .69] |  |  |
|  | Factor 1 | 0.15 | 0.05 | 2.82 | .01** | 1,16 [1.05 – 1.29] | 0.074 |  |
|  | Factor 2 | -0.07 | 0.05 | -1.34 | 0.18 | .94 [.84 – 1.03] |  |  |
|  | Intellectual Disabilities | -0.75 | 0.26 | -2.86 | .01* | .47 [.28 - .79] |  |  |
|  | | | | | | |  |  |
| *(c) Missing data analysis, Multinomial logistic regression: PCL:SV and ward location at 12 month follow up* | | | | | | |  |  |
|  |  |  |  |  |  |  |  |  |
|  |  | Location | β | SE | Z | *p* | OR (95% CI) | Pseudo R^2^ (McFaddon) |
| Model 1 | PCL:SV Total | Transferred | -0.02 | 0.03 | -0.57 | 0.57 | .98 [.93 - .1.04] | 0.051 |
|  |  | Discharged | -0.13 | 0.03 | -4.46 | <.001*** | .87 [.82 - .93] |  |
|  | Intellectual Disabilities | Transferred | -0.17 | 0.32 | -0.54 | 0.59 | .84 [.45 - 1.57] |  |
|  |  | Discharged | 0.31 | 0.31 | 1.01 | 0.31 | 1.36 [.75 - 2.48] |  |
| Model 2 | Factor 1 | Transferred | -0.11 | 0.07 | -1.67 | 0.1 | .99 [.87 - 1.13] | 0.052 |
|  |  | Discharged | -0.01 | 0.07 | -0.1 | 0.92 | 0.89 [.78 - 1.02] |  |
|  | Factor 2 | Transferred | -0.14 | 0.06 | -2.27 | 0.03* | .97 [.86 - 1.10] |  |
|  |  | Discharged | -0.03 | 0.06 | -0.41 | 0.68 | 0.87 [.77 - .98] |  |
|  | Intellectual Disabilities | Transferred | 0.32 | 0.31 | 1.02 | 0.31 | .86 [.45 - 1.63] |  |
|  |  | Discharged | -0.16 | 0.33 | -0.49 | 0.63 | 1.37 [.74 - 2.56] |  |
|  |  |  |  |  |  |  |  |  |
| *(d) Missing data analysis, Logistic regression: PCL:SV and changes in security ward at 12 months.* | | | | | | |  |  |
|  |  |  |  |  |  |  |  |  |
|  |  |  |  |  |  |  |  |  |
|  |  | β | SE | z | *p* | OR (95% CI) | Pseudo R^2^ (McFaddon) |  |
| Changes in security ward | PCL:SV Total | 0.1 | 0.03 | 3.14 | <.01** | 1.10 (1.03 – 1.17) | 0.061 |  |
|  | Intellectual Disabilities | -0.26 | 0.31 | -0.83 | 0.41 | 0.77 [0.42 – 1.43] |  |  |
|  | Factor 1 | 0.12 | 0.06 | 2.09 | .04* | 1.12 (1.00 – 1.26) | 0.044 |  |
|  | Intellectual Disabilities | -0.19 | 0.31 | -0.6 | 0.55 | .83 [0.45 – 1.54] |  |  |
|  | Factor 2 | 0.17 | 0.06 | 2.83 | <.01** | 1.18 (1.04 – 1.33) | 0.065 |  |
|  | Intellectual Disabilities | -0.36 | 0.31 | -1.18 | 0.24 | .69 [.37 – 1.29] |  |  |
|  |  |  |  |  |  |  |  |  |
| *Note: Significance level, p* < .05, p ** < .01, p ** < .001**** | | | | | | |  |  |
| *SE = standard error, IRR = incidence rate ratio, OR = odds ratio,* | | | | | | |  |  |
| *CLoS = current length of stay (days), SPC = secure psychiatric care, CCR = convictions, cautions and reprimands, PD = personality disorder* | | | | | | |  |  |
| *Reference categories logistic regressions (PCL:SV and characteristics associated with psychopathy) = No forensic background, no diagnosis of PD, forensic section* | | | | | | |  |  |
| *Location in secure psychiatric hospital based on changes from location at baseline (no change, transferred, discharge)* | | | | | | |  |  |
| *Reference category for logistic regression (changes in security ward) = no change* | | | | | | |  |  |

| **Table 9** | | | | | | | | |  |
| --- | --- | --- | --- | --- | --- | --- | --- | --- | --- |
| *Missing data analysis, Logistic regression and AUC analysis: PCL:SV scores as predictors of aggressive/ problematic behaviours at 12 months* | | | | | | | | |  |
|  |  | Β | SE | z | *p* | OR (95% CI) | AUC (95% CI) | AUC *p* value |  |
| Physical Aggression | PCL:SV Total | 0.01 | 0.03 | 0.16 | 0.87 | 1.01 [.94 - 1.07] | 0.55 [0.45 -0.64] | 0.16 |  |
|  | Intellectual Disabilities | 0.23 | 0.28 | 0.81 | 0.42 | 1.26 [.71 - 2.21] |  |  |  |
|  | Factor 1 | -0.04 | 0.04 | -0.9 | 0.37 | 0.96 [.37 - .88] | 0.54 [0.45 - 0.63] | 0.18 |  |
|  | Intellectual Disabilities | 0.18 | 0.29 | 0.64 | 0.53 | 1.20 [.53 - .53] |  |  |  |
|  | Factor 2 | 0.05 | 0.06 | 0.79 | 0.45 | 1.05 [0.92 - 1.19] | 0.58 [0.49 - 0.67] | 0.04* |  |
|  | Intellectual Disabilities | 0.21 | 0.28 | 0.74 | 0.46 | 1.23 [0.70 - 2.16] |  |  |  |
| Verbal Aggression | PCL:SV Total | 0.08 | 0.03 | 2.55 | 0.02* | 1.08 [1.01 - 1.15] | 0.65 [ 0.57 - 0.73] | <.001*** |  |
|  | Intellectual Disabilities | -0.06 | 0.33 | -0.17 | 0.86 | 0.94 [0.47 - 1.91] |  |  |  |
|  | Factor 1 | 0.12 | 0.06 | 2.08 | 0.06 | 1.12 [0.06 - 1.00] | 0.61 [0.53 - 0.70] | <.01** |  |
|  | Intellectual Disabilities | 0.01 | 0.34 | 0.03 | 0.98 | 1.01 [0.50 - 2.05] |  |  |  |
|  | Factor 2 | 0.14 | 0.05 | 2.6 | 0.22 | 1.15 [1.02 - 1.30] | 0.66 [0.58 - 0.74] | <.001*** |  |
|  | Intellectual Disabilities | -0.16 | 0.32 | -0.49 | 0.63 | 0.86 [0.44 - 1.67] |  |  |  |
| Sexual Behaviour | PCL:SV Total | 0.07 | 0.03 | 2.09 | 0.05 | 1.07 [1.00 - 1.14] | 0.66 [0.57 - 0.76] | <.01** |  |
|  | Intellectual Disabilities | 0.01 | 0.33 | 0.03 | 0.97 | 1.01 [0.53 -1.93] |  |  |  |
|  | Factor 1 | 0.09 | 0.01 | 1.46 | 0.17 | 1.10 [0.96 -1.25] | 0.64 [0.54 - 0.75] | <.01** |  |
|  | Intellectual Disabilities | 0.06 | 0.33 | 0.19 | 0.85 | 1.06 [0.55 2.05] |  |  |  |
|  | Factor 2 | 0.12 | 0.06 | 2.07 | 0.05 | 1.13 [1.00 - 1.28] | 0.66 [.56 -.76] | <.01** |  |
|  | Intellectual Disabilities | -0.08 | 0.33 | -0.23 | 0.82 | 0.93 [0.48 - 1.78] |  |  |  |
| Violence Towards Self | PCL:SV Total | 0.02 | 0.03 | 0.59 | 0.56 | 1.02 [0.95 - 1.09] | .65 [.55 - .75] | <.01** |  |
|  | Intellectual Disabilities | 0.54 | 0.34 | 1.59 | 0.12 | 1.71 [.86 - 3.40] |  |  |  |
|  | Factor 1 | 0.01 | 0.05 | 0.1 | 0.92 | 1.00 [0.91 - 1.11] | 0.65 [.55 - .75] | <.01** |  |
|  | Intellectual Disabilities | 0.53 | 0.35 | 1.53 | 0.14 | 1.70 [0.84 - 3.46] |  |  |  |
|  | Factor 2 | 0.06 | 0.06 | 1.05 | 0.31 | 1.06 [0.94 - 1.21] | 0.65 [0.55 - 0.75] | <.01** |  |
|  | Intellectual Disabilities | 0.51 | 0.35 | 1.46 | 0.16 | 1.66 [0.82 - 3.38] |  |  |  |
| Rule Breaking | PCL:SV Total | 0.03 | 0.03 | 1.24 | 0.23 | 1.03 [0.98 - 1.09] | 0.64 [0.55 - 0.72] | <0.01** |  |
|  | Intellectual Disabilities | 0.41 | 0.31 | 1.34 | 0.19 | 1.51 [0.81 - 2.82] |  |  |  |
|  | Factor 1 | 0.05 | 0.05 | 0.05 | 1.1 | 1.05 [0.96 - 1.16] | 0.64 [0.55 - .72] | <.01** |  |
|  | Intellectual Disabilities | 0.44 | 0.44 | 0.31 | 1.44 | 1.56 [0.83 - 2.92] |  |  |  |
|  | Factor 2 | 0.06 | 0.06 | 1.11 | 0.29 | 1.07 [0.94 - 1.20] | 0.64 [0.55 - 0.72] | <.01** |  |
|  | Intellectual Disabilities | 0.37 | 0.31 | 1.17 | 0.25 | 1.44 [0.76 - 2.75] |  |  |  |
| Threats of Aggression | PCL:SV Total | 0.06 | 0.04 | 1.74 | 0.11 | 1.06 [0.98 - 1.15] | 0.56 [.46 - .65] | 0.14 |  |
|  | Intellectual Disabilities | -0.02 | 0.31 | -0.07 | 0.94 | 0.98 [0.52 - 1.83] |  |  |  |
|  | Factor 1 | 0.09 | 0.06 | 1.56 | 0.14 | 1.10 [0.96 - 1.25] | 0.56 [.46 - .65] | 0.14 |  |
|  | Intellectual Disabilities | 0.04 | 0.32 | 0.11 | 0.91 | 1.04 [0.55 - 1.96] |  |  |  |
|  | Factor 2 | 0.12 | 0.06 | 2.03 | 0.06 | 1.13 [0.99 - 1.28] | .56 [.46 - .65] | 0.14 |  |
|  | Intellectual Disabilities | -0.11 | 0.32 | -0.32 | 0.74 | 0.90 [0.47 - 1.72] |  |  |  |
| Intimidating Behaviour | PCL:SV Total | 0.07 | 0.03 | 2.49 | 0.02 | 1.07 [1.01 - 1.13] | .50 [.41 - .59] | 0.49 |  |
|  | Intellectual Disabilities | -0.08 | 0.36 | -0.23 | 0.82 | 0.92 [0.43 - 1.98] |  |  |  |
|  | Factor 1 | 0.09 | 0.04 | 2.03 | 0.04 | 1.09 [1.00 - 1.19] | .50 [.41 - .59] | 0.49 |  |
|  | Intellectual Disabilities | -0.03 | 0.37 | -0.09 | 0.93 | 0.97 [0.44 - 2.14] |  |  |  |
|  | Factor 2 | 0.13 | 0.05 | 2.5 | 0.02 | 1.14 [1.02 - 1.27] | .50 [.41 - .59] | 0.49 |  |
|  | Intellectual Disabilities | -0.18 | 0.37 | -0.47 | 0.64 | 0.84 [0.38 - 1.85] |  |  |  |
| Inappropriate Behaviour | PCL:SV Total | 0.01 | 0.03 | 0.33 | 0.75 | 1.01 [0.94 - 1.08] | .61 [.49 - .73] | 0.025 |  |
|  | Intellectual Disabilities | 0.52 | 0.38 | 1.38 | 0.18 | 1.68 [0.76 -3.71] |  |  |  |
|  | Factor 1 | 0.01 | 0.06 | 0.23 | 0.82 | 1.01 [.89 -1.15] | .61 [.49 - .73] | 0.025 |  |
|  | Intellectual Disabilities | 0.53 | 0.39 | 1.37 | 0.19 | 1.70 [.76 - 3.83] |  |  |  |
|  | Factor 2 | 0.03 | 0.06 | 0.5 | 0.64 | 1.03 [.90 - 1.17] | 0.61 [.49 - .73] | 0.025 |  |
|  | Intellectual Disabilities | 0.5 | 0.38 | 1.32 | 0.2 | 1.65 [.74 - 3.68] |  |  |  |
| Overall Presence | PCL:SV Total | 0.06 | 0.03 | 2.5 | 0.01 | 1.07 [1.01 - 1.12] | .6 [.5 - .70] | 0.02* |  |
|  | Intellectual Disabilities | 0.47 | 0.43 | 1.08 | 0.31 | 1.60 [.6 -4.22] |  |  |  |
|  | Factor 1 | 0.08 | 0.06 | 1.33 | 0.2 | 1.08 [.95 - 1.23] | .60 [.50 - .60] | 0.02* |  |
|  | Intellectual Disabilities | 0.5 | 0.42 | 1.19 | 0.26 | 1.65 [.65 - 4.24] |  |  |  |
|  | Factor 2 | 0.12 | 0.05 | 2.36 | 0.03 | 1.13 [1.01 - 1.26] | .6 [.5 - .70] | .02* |  |
|  | Intellectual Disabilities | 0.39 | 0.44 | 0.9 | 0.39 | 1.48 [.55 - 3.95] |  |  |  |
| Violent Intent | PCL:SV Total | 0.08 | 0.02 | 3.16 | <.01** | 1.08 [1,03 - 1.13] | .61 [53 -0.7] | <.01** |  |
|  |  |  |  |  |  |  |  |  |  |
|  | Intellectual Disabilities | 0.52 | 0.45 | 1.16 | 0.28 | 1.68 [.60 - 4.72] |  |  |  |
|  | Factor 1 | 0.06 | 0.05 | 1.28 | 0.21 | 1.06 [0.97 - 1.16] | .61 [.53 - .7] | <.01** |  |
|  | Intellectual Disabilities | 0.51 | 0.44 | 1.16 | 0.28 | 1.67 [0.60 - 4.68] |  |  |  |
|  | Factor 2 | 0.18 | 0.05 | 3.97 | <.001*** | 1.20 [1.09 - 1.31] | .61 [.53 -.7] | <.01** |  |
|  | Intellectual Disabilities | 0.42 | 0.45 | 0.95 | 0.37 | 1.53 [.54 - 4.29] |  |  |  |
| *Note: Significance level, p* < .05, p ** < .01, p *** < .001, SE = standard error, CI = confidence intervals,* | | | | | | | | |  |
| *Reference category = behaviour not present* | | | | | | | | |  |
